# Lymphoma monocyte crosstalk via HSP27 to promote immune suppression and chemotherapy resistance

**DOI:** 10.1186/2051-1426-2-S3-P222

**Published:** 2014-11-06

**Authors:** Zhe Zhang, Peggy Bulur, Michael Gustafson, Dennis Gastineau, Allan Dietz, Yi Lin

**Affiliations:** 1 Division of Hematology, Dept of Medicine, Mayo Clinic, Rochester, MN, USA; 2 Division of Transfusion Medicine, Dept of Lab Medicine and Pathology, Mayo Clinic, Rochester, MN, USA

## Background

Immune editing is a major mechanism used by tumors to promote its survival. We have reported previously the presence of a novel phenotype of immunosuppressive monocytes (CD14^+^HLA-DR^low/neg^) in a number of cancers. Increased presence of these cells was associated with decreased treatment response and OS. We have demonstrated that certain tumor cells can convert normal CD14^+^HLA-DR^+ ^monocytes to CD14^+^HLA-DR^low/neg ^phenotype in an IL-10 independent and a tumor specific way. These CD14^+^HLA-DR^low/neg ^monocytes, in turn, protect tumors from cytotoxic killing from chemotherapy. Here we report up-regulation of heat shock protein-27 (HSP27) as one mechanism mediating this effect.

## Method

Monocytes from healthy donors were co-cultured with lymphoma cell lines (OCI-Ly3, Jeko-1, and Granta-519) with or without Doxorubicin (DOX). Cultured cells were assessed for phenotype, viability and proliferation by flow cytometry. Lymphoma cells were isolated with anti-CD19 immunomagnetic beads and assayed by immunoblot for expressions of proteins regulating apoptosis. HSP27 levels in human plasma were measured by ELISA.

## Results

DOX incubation induced apoptosis and decreased viability of all three cell lines; and co-culture with monocytes improved the lymphoma cell survival (for example, untreated Granta-519 had a 2.1 ± 0.45 fold expansion, that was reduced to 0.39 ± 0.12 when treated with DOX and 0.81 ± 0.27 after co-culture with monocytes with DOX. p < 0.05, n = 11.) Co-culture with monocytes induced increased HSP27 expression in lymphoma cells. HSP27 levels were further increased in co-culture with monocytes and DOX, with corresponding decrease in cleaved Caspase-3 levels. As tumor cells can secrete HSP27, we found detectable levels of HSP27 in plasma of lymphoma patients. Increased HSP27 in plasma correlated with increased proportion of CD14^+^HLA-DR^low/neg ^monocytes in blood.

## Conclusions

We have found that monocytes may promote lymphoma resistance to DOX killing by inducing increased HSP27. In turn, HSP27 from lymphoma patients may induce immune suppressive phenotype in monocytes. Together, this data demonstrates an active cross talk between monocytes and lymphoma resulting in multiple mechanisms of tumor resistance to chemo-immunotherapy.

